# Adaptive responses of *Trichlorobacter lovleyi* to nitrite detoxification reveal overlooked contributions of *Geobacterales* to nitrate ammonification

**DOI:** 10.1093/ismejo/wraf054

**Published:** 2025-03-18

**Authors:** Marcela Tabares, Kazem Kashefi, Gemma Reguera

**Affiliations:** Department of Microbiology, Genetics & Immunology, Michigan State University, East Lansing, MI 48824, United States; Department of Microbiology, Genetics & Immunology, Michigan State University, East Lansing, MI 48824, United States; Department of Microbiology, Genetics & Immunology, Michigan State University, East Lansing, MI 48824, United States

**Keywords:** DNRA, dissimilatory reduction of nitrate to ammonium, global nitrogen conservation, paddy soils, riparian corridors, nitrate reduction, nitrite toxicity

## Abstract

Poorly understood microorganisms “short-circuit” the nitrogen cycle via the dissimilatory nitrate reduction to ammonium to retain the element in agricultural lands and stimulate crop productivity. The prevalence of *Geobacterales* closely related to *Trichlorobacter lovleyi* in nitrate ammonification hotspots motivated us to investigate adaptive responses contributing to ammonification rates in the laboratory type strain *T. lovleyi* SZ. Here, we describe the identification of tightly regulated pathways for efficient nitrate foraging and respiration with acetate, an important intermediate of organic matter degradation that *Geobacterales* efficiently assimilate and oxidize. Challenging the established dogma that high carbon/nitrate ratios stimulate the reduction of nitrate to ammonium, *T. lovleyi* doubled rapidly across a wide range of ratios provided nitrate concentrations were low enough to prevent the accumulation of the toxic nitrite intermediate. Yet, excess electrons during hydrogenotrophic growth alleviated nitrite toxicity and stimulated the reduction of nitrate to ammonium even under conditions of severe acetate limitation. These findings underscore the importance of nitrite toxicity in the ammonification of nitrate by *Geobacterales* and provide much needed mechanistic understanding of microbial adaptations contributing to soil nitrogen conservation. This information is critical to enhance the predictive value of genomic-based traits in environmental surveys and to guide strategies for sustainable management of nitrogen fertilization as well as mitigation of green-house emissions and agrochemical leaching from agricultural lands.

## Introduction

**Figure 1:**
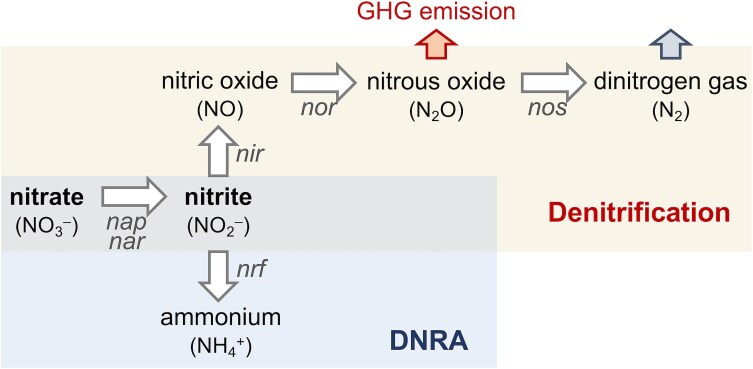
Microbial nitrate reduction pathways. Enzymatic reactions (associated genes in italics) for the microbial reduction of nitrate via denitrification (*top*) or ammonification (DNRA, *bottom*). The release of the greenhouse gas (GHG) nitrous oxide and the dinitrogen gas product during denitrification are also shown.

Sustainable management of nitrogen (N) in food and water systems requires innovative approaches to retain fixed N species and simultaneously stimulate crop productivity, reduce demands for inorganic fertilizers, and prevent N losses via gas emissions and leaching of mobile species [1]. Achieving these goals requires better understanding of the natural processes that promote N conservation in agricultural lands and the environmental conditions that control the microbial partitioning of N loss and retention pathways. The dissimilatory reduction of nitrate (NO_3_^−^) to ammonium (NH_4_^+^) via a nitrite (NO_2_^−^) intermediate [2, 3] (DNRA) is an attractive yet poorly understood process for the retention of soil N (Fig. 1). The lower (10–100 fold) diffusion coefficient of ammonium compared to nitrate reduces fertilizer mobility in soils and increases N availability to plants [4]. Nitrate may also be microbially reduced to gaseous N species via denitrification in a stepwise process that sequentially generates nitrite (NO_2_^−^), nitric oxide (NO), nitrous oxide (N_2_O, a greenhouse gas), and dinitrogen (N_2_) (Fig. 1). Denitrification is driven by facultative anaerobes when oxygen becomes limiting [5] and requires the metabolic integration of microorganisms with truncated denitrification pathways, which can reduce efficiency [6] but also enhance substrate consumption via cross-feeding of the toxic nitrite intermediate [7]. Oxygen levels also control the structure of denitrifying communities and their ability to use denitrification for the reductive detoxification of the NO intermediate [8]. Furthermore, an estimated 30% of denitrifying microorganisms lack enzymes for the last reductive step [9] and, thus, are a major source of greenhouse gas emissions [10]. Hence, there is significant interest in understanding the environmental partitioning of DNRA over denitrification as a strategy for N conservation, mitigation of greenhouse gas releases, and attenuation of N leaching from agricultural lands and runoffs.

Soil metagenomes sampled from around the globe contain a high representation of bacterial pathways for DNRA, suggesting important contributions to N conservation [11]. High carbon to nitrate (C/NO_3_^−^) ratio is often assumed to favor DNRA over denitrification [12–17]. However, bulk C/NO_3_^−^ calculations are not always a reliable predictor of the fate of nitrate [11, 18]. This is because many confounding variables such as climate, soil chemical or physical properties, plant community composition, and land-use history influence C/NO_3_^−^ and the selection of microbial pathways for nitrate reduction [19, 20]. Furthermore, the decomposition of organic matter increases the availability of carbon electron donors over nitrate, establishing conditions of nitrate limitation that could select for DNRA [21–23]. As a strong oxidizing agent [24], nitrate availability also influences the local redox potential and the partitioning of reductive pathways [1, 24], which typically favor DNRA under anoxic (thus more reducing) conditions [23].

Further limiting the environmental predictions, we currently lack well-defined laboratory representatives to mechanistically understand what variables control DNRA rates. Thermodynamics predicts lower theoretical energy yields from ammonification than denitrification (∆*G*^o^’ with glucose is −1796 kJ mol^−1^ and − 2669 kJ mol^−1^, respectively [25]). However, pure culture studies revealed similar [17] if not greater [26] growth yields and faster generation times [27] from ammonification when nitrate availability is low. This is because DNRA has a greater electron-accepting capacity compared to denitrification (8 electrons per nitrate instead of 5), making it a preferred sink for respiratory electrons when nitrate is the limited substrate [15, 27]. Bulk C/NO_3_^−^ measurements also fail to inform of the carbon type or whether non-carbon electron donors (e.g. H_2_ from the decomposition of organic matter) are present, which can greatly influence energy conservation from DNRA [26]. Other variables impacting the type and frequency of microbial pathways for N cycling, particularly soil C and temperature, make it difficult to identify lineages driving DNRA across different habitats [11]. Conservation of the first reductive step (nitrate to nitrite) in both ammonifiers and denitrifiers further limits genomic predictions (Fig. 1). Moreover, genes for the reduction of nitrite to ammonium (*nrf* genes) are broadly distributed among bacteria that do not necessarily grow via DNRA [11, 28]. Indeed, many *nrf*-containing taxa do not carry the genes for the reduction of nitrate to nitrite and/or may carry genes for some of the denitrification reactions [11]. Pure culture studies have also revealed the dual capacity of sulfite reductase enzymes to ammonify nitrite [29]. Further confounding the predictive power of the genomic traits, soil metagenomes show many genera containing both canonical pathways for DNRA and denitrification.

Some genera formerly within the class Deltaproteobacteria and recently reassigned [30] to the order *Geobacterales* (e.g. *Geobacter* and *Trichlorobacter*) are ubiquitous and highly represented in environments where DNRA is a significant process such as paddy soils and freshwater sediments along the edges of rivers, streams, lakes, and other water bodies (riparian corridors) [11, 28, 31–33]. Species abundance, however, cannot reliably predict DNRA rates in these environments, limiting the predictive value of the taxonomic analysis [31]. Furthermore, *Geobacterales* are often used as a proxy of metal reduction due to their ability to assemble complex and redundant respiratory chains of *c*-type cytochromes and retractable, conductive protein appendages of the Type IVa pilus subclass to efficiently discharge respiratory electrons onto the abundant iron (hydroxy)oxide (Fe_2_O_3_ • xH_2_O) minerals [34–36]. Yet, *Geobacterales* species, particularly those closely related to *Trichlorobacter lovleyi* (formerly *Geobacter lovleyi*), can be readily enriched and DNRA can be stimulated in these environments by lowering nitrate inputs [33]. Nitrogen-limited conditions also enrich for close relatives of *T. lovleyi* and partition DNRA over denitrification in chemostat cultures started with activated sludge [17]. These observations suggest critical roles for these bacteria in nitrate ammonifying communities as well as opportunities to target their DNRA metabolism for N management in agricultural soils. Such interventions build on the success of field trials previously used to control the mobility of metal contaminants by stimulating the *in situ* growth and metabolism of *Geobacterales* [37]. Leveraging the availability of a pure culture representative of the environmental *Geobacterales* enriched in DNRA sites (*T. lovleyi* SZ [38]), we established controlled laboratory conditions to investigate its nitrate-reducing physiology and mechanistically understand confounding variables influencing growth via nitrate ammonification. Results from this work identified genomic-based traits and adaptive responses that enhance the translational power of metadata to better predict overlooked contributions of *Geobacterales* to N retention across a wide range of C/NO_3_^−^ ratios. The results challenge the long-held generalization that a high C/NO_3_^−^ ratio is needed to promote DNRA and provide much needed understanding of microbial processes contributing to N conservation in agricultural lands.

## Materials and methods

### Bacterial strains and culture conditions

*T. lovleyi* SZ (ATCC BAA-1151; DSM 17278) was kindly donated by Dr. Dawn Holmes (University of Massachusetts, Amherst) and routinely cultured in anoxic DBAF, a bicarbonate-based mineral medium (DB) [39] supplemented with sodium acetate (20 mM) and sodium fumarate (40 mM) as the electron donor and acceptor, respectively. The medium (10 ml) was dispensed into “Balch-type” tubes (18 × 150 mm, Chemglass), sparged with an oxygen-free gas mix of N_2_:CO_2_ (80:20) for 20 min, sealed with 20-mm butyl rubber stoppers and aluminum seals (Chemglass), and autoclaved for 30 minutes. Cysteine•HCl (2 mM, final concentration) was added as a reducing agent from an autoclaved anoxic stock (20 mM) prior to inoculation with mid-exponential cells (10% optical density at 600 nm, OD_600_, of 0.2) and incubation at 30°C. When indicated, the concentration of CaCl_2_ in the DB media (120 mg/L) was reduced 10-fold (modified DB medium or mDB) to reportedly stimulate DNRA [40].

The DBAF cultures were transferred twice in mid-exponential phase before a third transfer (starting OD_600_ of 0.02) into fresh DB medium with 20 mM acetate and 5 mM nitrate (DBAN) or, when indicated, other electron donors (Table S1). For hydrogenotrophic growth studies, H_2_ was provided as an oxygen-free gas mix of H_2_:CO_2_ (80:20) in the culture headspace. The 20 mM acetate/5 mM nitrate cultures were transferred three times before inoculating (10% OD_600_ 0.2) cultures containing the same or higher concentration of nitrate (up to 30 mM) to provide molar ratios of C/NO_3_^−^ from 0.3 to 3. All cultures were incubated at 30°C and periodically monitored for growth (OD_600_) and nitrate ammonification (chemical detection of N species in stationary phase cultures, as described below).

### Analytical techniques

Organic acids were measured in filtered (0.45 μm syringe filters, Thermo Scientific Titan3) stationary-phase culture supernatant fluids by high-performance liquid chromatography (HPLC) (Waters, Milford, MA) using a 300 mm × 7.8 mm Aminex HPX-87H column (Bio-Rad), as reported elsewhere [39]. Nitrate and nitrite were also measured by HPLC in a Shimatzu LC-20 AD instrument with a protocol based on two other methods [41, 42]. Briefly, nitrate and nitrite in filtered supernatant fluids were separated in a 3.9 mm × 7.8 mm C18 column (Waters) at a flow rate of 0.6 ml/min using a mobile phase of 10 mM octylamine and 5 mM tetrabutylammonium hydrogen sulfate (pH 6.5, adjusted with phosphoric acid). Detection was with a UV detector at a wavelength of 210 nm using aqueous solutions of sodium nitrate and sodium nitrite as standards. Ammonium in the samples was measured with a colorimetric enzyme-based ammonia assay kit (MilliPore Sigma) after subtracting the ammonium detected in uninoculated media controls (~3.7 mM) and in the 15 mM acetate/5 mM nitrate inoculum (~0.5 mM).

### RNA-Seq

We extracted RNA from triplicate 20 mM acetate DB cultures transferred three times and grown to late exponential phase (~0.18 OD_600_) with 5 mM nitrite or 20 mM fumarate, which are conditions that supported similar growth yields after ~9 h of incubation at 30°C. RNA transcription was stopped with 10 ml of 5% (v/v) water-saturated phenol (pH 6.6 in ethanol; Ambion) before harvesting the cells by centrifugation (5000 rpm, 8 min, 4°C) and extracting the RNA with the QIAGEN RNeasy kit. DNA digestion with RNase-free DNase Set (QIAGEN) followed manufacturer’s recommendations. RNA purity was confirmed by reverse transcription-PCR (RT-PCR) with Verso 1-step RT-PCR kit (Ambion) and, when needed, after a second DNase treatment to remove any residual DNA from the sample. RNA integrity was confirmed using a 2100 BioAnalyzer (Agilent). The steps of rRNA depletion, library preparation, and sequencing on a Hi-Seq 4000 System (Illumina) followed standard protocols at Michigan State University Research Technology Support Facility using the TruSeq Total RNA Library Preparation Kit with RiboZero Bacteria rRNA depletion beads (Illumina). Completed libraries were QC’d and quantified using a combination of Qubit dsDNA HS and Advanced Analytical Fragment Analyzer High Sensitivity DNA NGS assays. All libraries were pooled in equimolar amounts for multiplexed sequencing. The pool was quantified using the Kapa Biosystems Library Quantification qPCR kit (Illumina). This pool was loaded onto one lane of an HiSeq 4000 flow cell for sequencing (1x50bp single read) using HiSeq 4000 SBS reagents (Illumina). Base calling was done by Real Time Analysis (RTA) v2.7.7 (Illumina) and output of RTA was demultiplexed and converted to FastQ format with Bcl2fastq v2.19.1 (Illumina). RNA-Seq data processing and analysis was through SPARTA platform [43] and it included: quality control and trimming with FastQC and Trimmomatic tools, respectively; RNA alignment against the reference *T. lovleyi* SZ genome (CP001089.1) with bowtie tool; gene level quantification of the aligned sequences with the HTSeq software package; and differential gene expression by normalizing the data with edgeR tool. The final values were filtered by FDR (false discovery rate, < 0.05) and Log CPM (counts per million, >5), and by Log FC (fold change) of <−1 or > 1 to obtain the downregulated and upregulated genes, respectively. Visualization was with a heatmap generated using the *pheatmap* function in the R software [44].

### Quantitative reverse transcription polymerase chain reaction

We compared the expression of *T. lovleyi* SZ genes encoding nitrate reductases (*napA*, *narG*-1, *narG*-2) and nitrite reductases (*nrfA*-1, *nrfA* − 2, *nrfA* − 3) in DBAN cultures grown with 20 mM acetate electron donor and either 5 mM (optimal growth) or 10 mM (suboptimal growth) of nitrate as the electron acceptor. As control, we used the *rec*A gene, a single-copy gene that is constitutively expressed in *Geobacteraceae* [45, 46]. Primers for the reverse transcription polymerase chain reaction analysis are listed in Table S2. RNA extraction and DNase treatment were as described for the RNA-Seq experiments. The RNA served as template for cDNA synthesis with the RevertAid First Strand cDNA Synthesis Kit (ThermoFisher Scientific). This reaction provided the cDNA template used for the amplification of targeted genes in a 10 μl qPCR reaction containing 5 μl of 10x SsoAdvanced universal SYBR green supermix (Bio-Rad), 250–500 nM of each primer, and 100 ng of cDNA. Amplification was in a CFX96 Touch Real -Time PCR Detection system (Bio-Rad) using cDNA denaturation at 95°C for 30 sec, 35 amplification cycles with denaturation at 95°C for 15 sec, and annealing extension at 52°C for 30 sec. Amplicon size was confirmed electrophoretically in an agarose (1%) gel. Standard curves for quantitative analysis were prepared as described elsewhere [46].

### Nitrate/nitrite reductase assays

The enzymatic reduction of nitrate and nitrite was measured in protein extracts from triplicate 50-ml cultures (20/5 and 30/10 mM concentrations of acetate/nitrate) of *T. lovleyi* SZ using a published methyl viologen spectrophotometric assay [47]. Cells were harvested by centrifugation (13 000 rpm, 5 min, 4°C) after ~9 h (20/5 cultures) or ~ 40 h (30/10 cultures) of growth at 30°C and resuspended in 5 μl of 10X phosphate buffered saline (PBS) prior to 5 cycles of sonication in a Sonifier 450 (Branson), each with a 30% duty cycle. Output control started at 1 and increased to 3 over time, with each cycle lasting 10 seconds. Throughout the process, samples were kept on ice. Cell extracts were storage at −20°C until ready for use. The reaction mix (1 ml) was prepared in a sealed quartz cuvette (Sutaluna Quartz Cell) under anoxic conditions (COY-Labs glove chamber with an anoxic atmosphere of 83:10: 7 N_2_:CO_2_:H_2_) and contained sodium bicarbonate buffer (10 mM), nitrate or nitrite as terminal electron acceptor (5 mM), dithionite (reducing agent, 5 mM), methyl viologen (electron donor, 4 mM), and protein extract (2 μg). Controls without or with denatured (100°C, 10 min) protein extract, or controls without electron acceptor (nitrate or nitrite), were also included. Cuvettes with the reaction mixture were sealed in the glove bag and incubated at 30°C inside a UV-2600 spectrophotometer (Shimadzu) to monitor at 600 nm the disappearance of the blue coloration caused by the oxidation of the reduced methyl viologen during the reduction of nitrate or nitrite.

### Phylogenetic analysis of NrfA proteins

Phylogenetic analysis of the NrfA proteins of *T. lovleyi* SZ was as described previously [48] and used 44 closely related bacterial NrfA amino acid sequences retrieved from FunGene [49] and aligned with MUSCLE [50]. Trimming was used to preserve the sequences containing the third and fourth conserved heme-binding motifs (CXXCH) and the arginine residue for Ca^2+^-independent (XXRH) or Ca^2+^-dependent (KXQH) motifs. Bayesian analysis of the 44 sequences was in MEGAX [51, 52] following the model LG + G + I, maximum likelihood model selection and 500 bootstrap replications [48]. The FigTree v1.4.4 program (http://tree.bio.ed.ac.uk/software/figtree/) was used for post-processing and tree visualization.

## Results

### Genomic-based predictors of *T. lovleyi* contributions to DNRA

**Figure 2:**
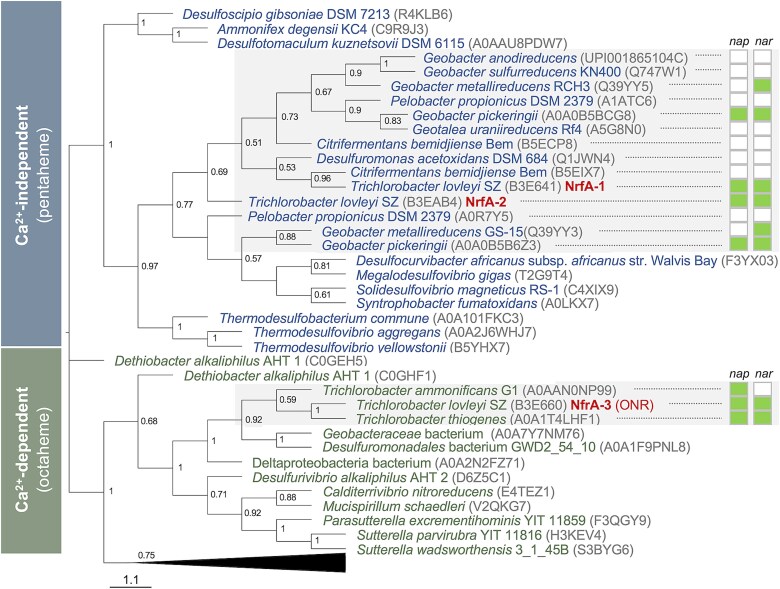
Nitrite reductases of *T. lovleyi* and close relatives*.* Maximum likelihood phylogenetic tree of the *T. lovleyi* nitrite reductase (NrfA) enzymes and closely related bacterial NrfA proteins within the *Geobacterales* (shaded in gray) and other orders. The inset at right shows the nitrate reductase inventory (Nap or Nar systems) for *Geobacteraceae* species and close relatives in the *Desulfuromonas* and *Pelobacter* genera. The tree shows the separate clustering of the *T. lovleyi* SZ NrfA-1 and NrfA-2 enzymes with other Ca^2+^-independent pentaheme NrfA proteins and the independent clustering of NrfA-3 with Ca^2+^-dependent, octaheme nitrite reductase (ONR) proteins. The collapsed outgroup cluster includes the Ca^2+^-dependent pentaheme NrfA enzymes of *Wolinella succinogenes*, *Escherichia coli*, *Desulfovibrio vulgaris*, and *Sulfurospirillum deleyianum*. Amino acid sequences were aligned, and the tree was constructed using the MEGA v 10.1 X software, as described before [48] (scale bar, substitutions per site; bootstrap values >0.5 are shown on top of each branch).

The order *Geobacterales* includes species formerly assigned to the single genus *Geobacter* within the family *Geobacteraceae* and now reclassified [30] into various genera within this and the *Pseudopelobacteraceae* family. Over a third of pure culture representatives, including *T. lovleyi* SZ, are reportedly capable of DNRA growth with nitrate using acetate as the electron donor [37]. This finding suggested a widespread capacity of the group to ammonify nitrate with their preferred electron donor, acetate [37]. The sequenced genomes also predict a widespread ability of the group to enzymatically reduce the nitrite intermediate via pentaheme nitrite reductases (NrfA) independently evolved for Ca^2+^-independent reduction of nitrite to ammonium [48] (Fig. 2). *T. lovleyi* SZ encodes two of these pentaheme NrfA proteins (NrfA-1 and NrfA-2) as well as an octaheme nitrite reductase (ONR) enzyme (NrfA-3) with the conserved active site of Ca^2+^-dependent enzymes (Fig. 2). This contrasts with *Trichlorobacter ammonificans,* a close relative of *T. lovleyi* isolated from activated sludge in nitrate-limited chemostats with acetate [53], which encodes the ONR but no pentaheme NrfA enzymes (Fig. 2). Thus, *T. lovleyi* is unique in being equipped with three rather than one nitrite reductase enzyme for the ammonification of nitrite.

In *T. ammonificans*, ONR partners with a Nap nitrate reductase to reduce nitrate to ammonium in the periplasm [53]. *T. lovleyi* SZ genome also encodes a Nap system but it is one of a few pure culture representatives with Nar complexes (two complete and one incomplete) for the cytoplasmic reduction of nitrate in a reaction that generates protons for ATP synthesis [54] (Fig. 2). Redundant pathways for nitrate and/or nitrite reduction are not uncommon in the *Geobacteraceae*, whose gene inventory splits the family into complete and partial ammonifiers [55]*.* For example, the best studied member of the *Geobacteraceae*, *Geobacter sulfurreducens,* is a partial ammonifier: its genome encodes a Ca^2+^-independent pentaheme NrfA for the reduction of nitrite to ammonium but lacks nitrate reductase enzymes (Fig. 2). As a result, it cannot grow via DNRA with nitrate [56]. However, it can reduce nitrite to ammonium at a wide range of C/N ratios [57] and it can scavenge the intermediate from denitrifying communities to stimulate denitrification [56]. The nitrite ammonification reaction of *G. sulfurreducens* is also faster than in complete ammonifiers such as *Geobacter metallireducens* and *T. lovleyi* [57] despite the presence in both organisms of more than one pentaheme NrfA enzymes (Fig. 2). Kinetic data also indicates a more favorable reduction of nitrate to nitrite in *T. lovleyi* compared to *G. metallireducens* [57], possibly due to the presence of both Nap and Nar complexes in the former and only Nar in the latter (Fig. 2). This could explain the prevalence of close relatives of *T. lovleyi*, but not of *G. metallireducens*, in DNRA hotspots and enrichments. The genomic and biochemical data thus suggest widespread contributions of *Geobacterales* to the ammonification of nitrate and/or nitrite, but enzymatic adaptations in *T. lovleyi* that could determine its ecological fitness in DNRA-active communities.

### Transcriptional acclimation of *T. lovleyi* to DNRA growth

**Figure 3:**
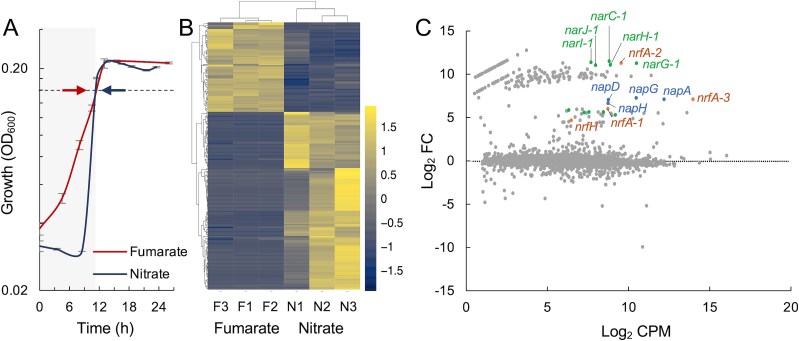
Genes differentially expressed with nitrate versus fumarate. (A) Growth of *G. lovleyi* SZ with 20 mM acetate and 5 M nitrate or 20 mM fumarate as electron acceptor. Arrows show the point (OD_600_, ca. 0.18) of cell harvesting for RNA extraction and mRNA sequencing (RNA-Seq). (B-C) Heatmap (B) and dispersion plot (C) of genes differentially upregulated or downregulated in triplicate cultures with nitrate (N) versus fumarate (F). The dispersion plot shows the log of transcript counts per million (CPM, >5) versus the log of fold change (FC, <−1 or > 1) for differentially expressed genes with a false discovery rate FDR < 0.05. The plot highlights upregulated genes encoding nitrate (*nar* and *nap*) or nitrite (*nrf*) reductases.

To better understand the physiology of *Geobacterales* in DNRA hotspots, we cultivated the model representative *T. lovleyi* SZ with nitrate as the sole electron acceptor using a bicarbonate-based medium pre-reduced with cysteine, which previously was shown to be needed for optimal growth with other electron acceptors [58]. Acetate was the only electron donor that supported growth and continuous (3 or more) culture passages with nitrate (Table S1). Other potential carbon electron donors such as pyruvate and lactate or supplementation of the acetate-nitrate cultures with formate, a high energy-yielding electron donor that is co-assimilated for carbon with acetate [39], increased the C/NO_3_^−^ yet failed to support growth and nitrate ammonification. Growth efficiency was highest in acetate-nitrate cultures where nitrate was provided at 5 mM concentrations (Table S1). Under these conditions, cells consistently experienced a lag phase of ~8 h, even after continuous passages in the same medium (Fig. 3A). But, once acclimated, cells doubled twice faster (1.93 ± 0.06 h) than in control cultures with fumarate (4.3 ± 0.4 h) (Fig. 3A). This is consistent with the higher standard redox potential versus the standard hydrogen electrode of the nitrate/ammonium pair (*E_0_’* = 363 mV) compared to fumarate/succinate (*E_0_’* = 31 mV) [59].

The reproducibility of the lag phase in the acetate-nitrate cultures even after several passages in the same medium suggested that DNRA is a respiratory process requiring regulated acclimation. To better understand this, we sequenced RNA transcripts (RNA-Seq) from late-exponential cells grown with 20 mM acetate and 5 mM nitrate, which correspond to a C/NO_3_^−^ ratio (molar ratio of 4, or 3.8 mg acetate per mg nitrate) previously shown to partition DNRA over denitrification and enrich for *T. lovleyi* in nitrate-limited chemostats [17]. As controls, we also extracted and sequenced RNA from 20 mM acetate cultures with fumarate concentrations (20 mM) that yielded the same cell biomass (OD_600_ ~ 0.2) as the 5 mM nitrate cultures after 12 h of incubation at 30°C (Fig. 3A). The RNA-Seq analysis identified distinct, reproducible patterns of DNRA activation and deactivation for 200 genes (Fig. 3B). Among the differentially regulated genes, 133 were upregulated and 67 were downregulated (Table S3). The most highly upregulated genes were those encoding redundant pathways for nitrate (*nap* and *nar*) and nitrite (*nrf*) reduction (Fig. 3C), but also flagellar motility and chemotaxis proteins, as well as signaling proteins with Per-Arnt-Sim (PAS) domains for nitrate sensing (Table S4). These results suggested key contributions of sensory and respiratory pathways to the regulated acclimation of *T. lovleyi* to DNRA growth.

A model reconstructing the DNRA respiratory chain of *T. lovleyi* (Fig. S1) predicted redundant pathways for the flow of electrons from the reduced menaquinone (MQH_2_) pool in the inner membrane to nitrate via one periplasmic (*nap*) and two cytoplasmic (*nar*-1 and *nar*-2) nitrate reductase systems. Only Nar systems support energy conservation from the reduction of nitrate to nitrite [54]. Also upregulated in the DNRA cultures were the two Ca^2+^-independent pentaheme nitrite reductases (NrfA-1 and NrfA-2) and the Ca^2+^-dependent ONR enzyme (NrfA-3). NrfA-1 and NrfA-2 enzymes shared 72% similarity and clustered separately with other Ca^2+^-independent cytochrome *c* nitrite reductases in the *Geobacteraceae* (Fig. 2). The ONR NrfA-3 protein had much lower sequence similarity (39–40%) with the other two NrfA enzymes and clustered separately in the phylogenetic tree, consistent with a distinct ancestry (Fig. 2). The ONR-encoding gene (*nrfA-3*) is also immediately downstream of the *nap* genes (Table S3). Such a clustered arrangement suggests the functional coupling of the Nap and ONR proteins for the stepwise reduction of nitrate to ammonium via nitrite in the periplasm, as reported in the close relative *T. ammonificans* [53]. Similarly, the pentaheme NrfA-2 enzyme is located immediately downstream of one of the upregulated nitrate reductase clusters (*nar-1*) (Table S4), thus suggesting catalytic coupling of the two reactions as well (Fig. S1). NrfA-1, in contrast, is encoded with a membrane-bound, tetraheme cytochrome *c* protein (NrfH) in an operon that is conserved among *Geobacterales*, including partial ammonifiers such as *G. sulfurreducens*, as well as *Desulfovibrio* species (Fig. S2). NrfH dimerizes and binds NrfA dimers to transfer electrons from the reduced menaquinone pool to nitrite in the periplasm [60, 61]. Additional information about the nitrate and nitrite reductase enzymes of *T. lovleyi* is included in the Supplementary Discussion.

### Nitrite toxicity, rather than C/NO_3_^−^ ratios, limits DNRA growth in *T. lovleyi*

**Figure 4:**
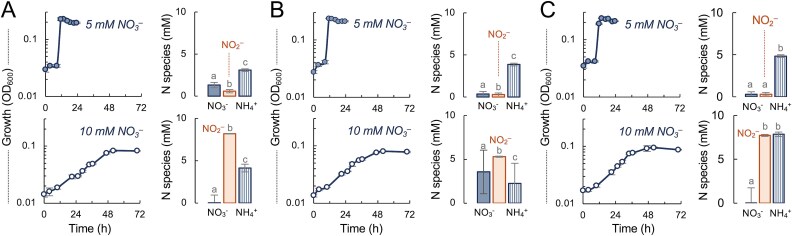
Effect of acetate and nitrate availability on DNRA growth. *G. lovleyi* SZ growth (optical density at 600 nm, OD_600_) and N species (nitrate, nitrite and ammonium) in cultures with 5 mM or 10 mM (open symbols) nitrate and acetate concentrations yielding molar C/NO_3_^−^ ratios of 1 (A), 2 (B), or 3 (C). The N species were measured in supernatant fluids recovered from early stationary phase cultures and differences for each treatment were statistically identified with a combined ANOVA and post-hoc Tukey HSD test (*a*, *b* or *c* for *P* < .05).

DNRA growth efficiency was high with 5 mM nitrate across molar C/NO_3_^−^ ratios covering conditions of acetate (C/NO_3_^−^ of 1) or nitrate (C/NO_3_^−^ of 2 or 3) limitation (Fig. 4). Acetate limitation prevented the complete reduction of the electron acceptor, converting 74% (3.9 ± 0.3 mM) of the available nitrate to nitrite (0.6 ± 0.2 mM) and ammonium (3.1 ± 0.3 mM) (Fig. 4A). This suggested that *T. lovleyi* diverted ~75% of acetate for its complete oxidation to CO_2_ in the tricarboxylic acid (TCA) cycle and generation of reducing power for nitrate reduction, using the remaining acetate for biomass synthesis. Higher concentrations of acetate to increase the molar C/NO_3_^−^ ratio in the 5 mM nitrate cultures from 1 to 2 and 3 alleviated electron donor limitation and promoted the recovery of practically all the nitrate as ammonium (Fig. 4B–C). The nitrite intermediate was also detected, albeit at levels <0.5 mM. By contrast, nitrite accumulated and growth rates and yields significantly decreased in cultures with 10 mM nitrate despite adjusting acetate concentrations to maintain the same C/NO_3_^−^ ratios (Fig. 4). Higher nitrate concentrations (20 mM) prevented growth, even after decreasing CaCl_2_ in the medium to reportedly stimulate nitrate reduction in the *Geobacteraceae* [40, 62] (Fig. S3). Chemical analysis of the N species in the 10 mM nitrate cultures demonstrated the reductive conversion of most of the nitrate to nitrite (~9 ± 3 mM, average for all treatments) (Fig. 4). Cells in these cultures were also inviable and could not be further propagated. We also detected in these cultures higher concentrations of ammonium than expected stoichiometrically, which is consistent with the detection in the enzymatic assay of protein and peptides released upon cell lysis. Inoculating the 10 mM nitrate media with cells adapted to growth with 5 mM nitrate did not improve the growth efficiency and cell viability in the cultures was similarly compromised.

**Figure 5:**
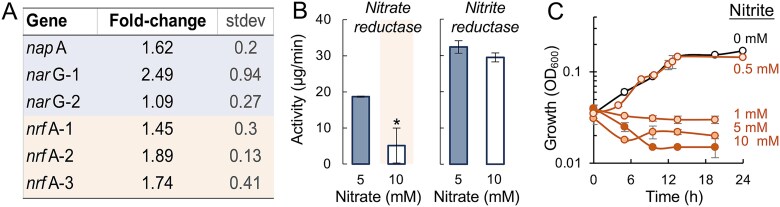
Nitrite toxicity limits growth via nitrate ammonification. (A) Fold-change increases in transcript levels for genes encoding nitrate reductases (*napA*, *narG*-1, *narG*-2), and nitrite reductases (*nrfA*-1, *nrfA* − 2, *nrfA* − 3) in 10 mM versus 5 mM nitrate cultures with 30 mM and 15 mM acetate (from Fig. 3C). Transcripts were measured by real time quantitative (RTq) PCR using *recA* transcripts as internal controls. (B) Nitrate and nitrite reductase activities (μg/min) in the 5- and 10-mM nitrate cultures measured as the rate of methyl viologen oxidation by culture cell extracts (2 μg of total protein) with 5 mM nitrate or nitrite as electron acceptor. c, average growth (optical density at 600 nm, OD_600_) in acetate-fumarate cultures with or without increasing concentrations of nitrite (0, 0.5, 1, 5, 10 mM). Shown are the average and standard deviation of triplicate cultures for each treatment. T-test was used to assessed statistical significance of the reductase activities in 5 versus 10 mM nitrate cultures is also shown (^*^P < .05).

Transcript measurements for key catalytic components of the DNRA respiratory chains confirmed their higher upregulation in the 10 mM than 5 mM nitrate cultures in reference to the housekeeping gene *recA* (Fig. 5A). Thus, cells acclimated to higher nitrate availability by transcriptionally upregulating the respiratory chains for both nitrate and nitrite reduction. However, the nitrate reductase enzyme activity was much lower and nitrite reductase activity did not increase in the 10 mM nitrate cultures compared to the 5 mM nitrate controls (Fig. 5B). The inhibition of nitrate reductase with 10 mM nitrate did not prevent nitrite from accumulating to levels (~8 mM) (Fig. 4C) well above cytotoxic thresholds (1 mM or higher) (Fig. 5C). The more favorable kinetics reported for nitrate than nitrite reduction in *T. lovleyi* [57] likely contributed to the rapid accumulation of nitrite. However, the results also support the notion that preferential pathways exist for diverting respiratory electrons from nitrite to nitrate reduction when nitrate concentrations are high, as reported in other bacteria [63, 64]. As a result, *T. lovleyi* cannot reductively detoxify nitrite in the 10 mM nitrate cultures before the intermediate accumulates to cytotoxic levels. This result underscores the importance of nitrite toxicity in controlling growth efficiency with nitrate and, by extent, DNRA rates.

### Excess electrons during hydrogenotrophic growth promote nitrite detoxification and stimulate nitrate ammonification

**Figure 6:**
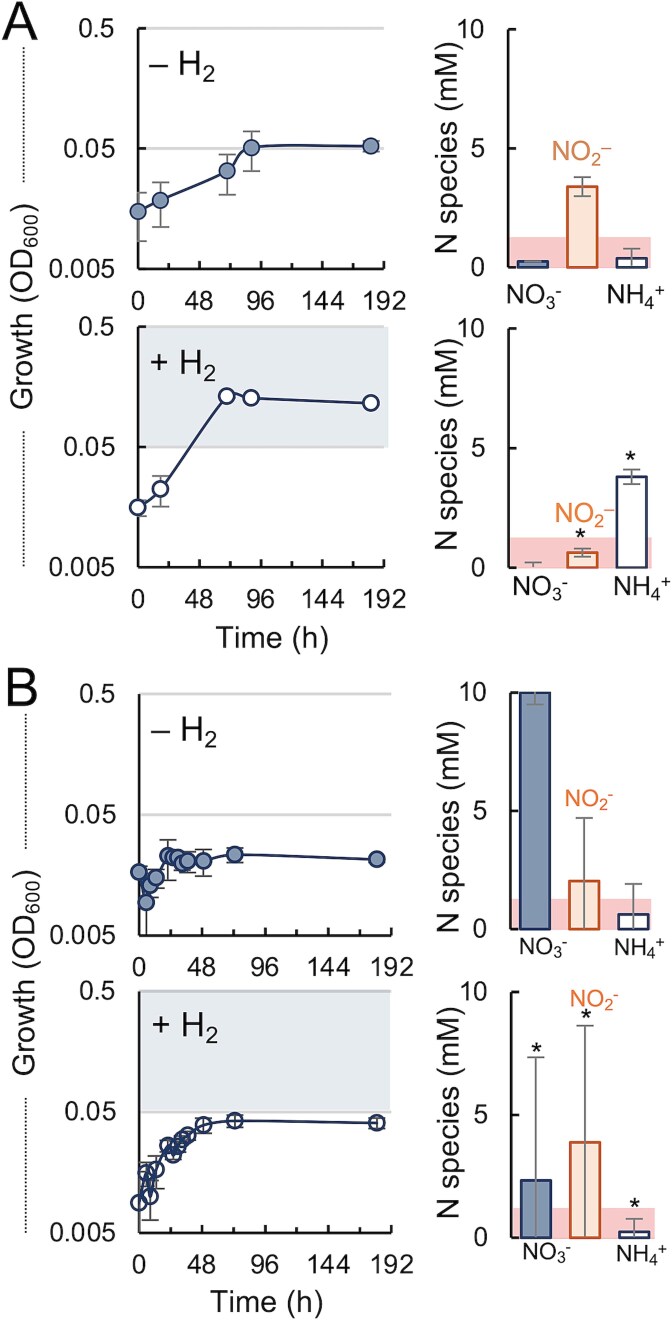
Hydrogenotrophic ammonification partially alleviates nitrite toxicity. Growth and N species (nitrate, nitrite and ammonium) detected in 5 mM (A) or 10 mM (B) nitrate cultures under exacerbated conditions of acetate limitation (3 mM acetate) with or without H_2_ available as an alternative electron donor. Statistically significant differences for each N species in the H_2_ cultures compared to the no-H_2_ controls were analyzed with an F-test to assess variance for each pairwise comparison followed by a 2-tail t-test (homoscedastic or heteroscedastic for equal or unequal variance, respectively) using the Microsoft Excel software (^*^P < .05).

Organic matter decomposition in DNRA hotspots generates H_2_, a high energy-yielding electron donor that stimulates the reductive activities of iron-reducing bacteria with acetate [65]. To test if H_2_ could provide excess electrons for the reductive detoxification of nitrite, we established culture conditions with 5 mM nitrate (3 mM acetate; molar C/NO_3_^−^ ratio of 0.6) that led to the accumulation of toxic levels of nitrite and, consequently, reduced growth efficiency (~55 h generation times, low cell yields) (Fig. 6A). As predicted, hydrogenotrophic conditions in these cultures provided excess electrons for the reductive detoxification of nitrite, and stimulated both growth (generation times ~26 h) and ammonium yields. Increasing nitrate concentrations from 5 to 10 mM in the 3 mM acetate cultures (molar C/NO_3_^−^ of 0.3) exacerbated nitrite toxicity and prevented growth with acetate unless in the presence of H_2_ (Fig. 6B). Hydrogenotrophic conditions in the severely acetate-limited cultures stimulated the reduction of nitrate to nitrite but did not support ammonium formation, consistent with the preferential diversion of respiratory electrons from nitrite towards nitrate reduction. Measurements of N species were also highly variable among the culture replicates, a metabolic heterogeneity typically displayed by populations adaptively responding to nutrient stress [66]. Thus, although hydrogenotrophic growth alleviated nitrite toxicity and stimulated DNRA in acetate-limited cultures, adequate levels of the electron donor and carbon source were needed for cells to effectively balance respiratory gains and nitrite detoxification.

## Discussion

Findings from this work challenge the value of C/NO_3_^−^ ratios to predict DNRA rates and support earlier work with pure cultures that predicted important contributions of *Geobacteraceae* to N retention even in organic limited soils [57]. We show, e.g. that *T. lovleyi* grew rapidly by ammonifying nitrate across a wide range of C/NO_3_^−^ ratios spanning conditions of acetate and nitrate limitation (Fig. 4). The rate-limiting step in all cases was the ability of the cells to prevent the accumulation of the nitrite intermediate above toxic concentrations (>0.5 mM) (Fig. 5). This result adds to a growing body of evidence contesting the reliability of bulk C/NO_3_^−^ measurements to predict soil DNRA [11, 18, 67, 68]. Indeed, the C/NO_3_^−^ ratio does not inform of carbon types, which can influence the frequency of individual N pathways [69] and whether the carbon substrate will be preferentially oxidized or fermented for DNRA [15]. Acetate, a non-fermentable substrate, was the only carbon electron donor that supported the growth of *T. lovleyi* SZ with nitrate under the study conditions (Table S1). The close relative, *T. ammonificans*, is also an acetate-dependent nitrate ammonifier isolated from sludge in ammonifying bioreactors but, unlike *T. lovleyi*, it lacks the canonical pentaheme NrfA pathways (Fig. 2) and solely relies on an ONR enzyme for DNRA [53]. The metabolic specialization towards acetate, the preferred electron donor and carbon source for *Geobacterales* [37], is not surprising given the abundance of this intermediate of organic matter degradation in anoxic environments where DNRA is an active process [65, 70]. Fertilization increases the concentration of nitrate in soils but also accelerates organic matter decomposition via denitrification, which reduces the labile fraction of soil organic carbon (SOC) [71–73] and increases acetate availability for DNRA [70]. Furthermore, *Geobacterales* express multiple transporters and oxidative pathways for acetate that ensure the efficient assimilation and oxidation of the substrate with a wide range of electron acceptors [37]. *Geobacterales* are also unique in their ability to divert most of the acetate for its complete oxidization to CO_2_ in the TCA cycle, which maximizes the generation of reducing power for ATP synthesis and respiratory growth [37]. We calculated that *T. lovleyi* diverted ~75% of the available acetate to ammonify nitrate. This is significantly higher than the reported efficiency of acetate oxidation (~60%) with fumarate, an electron acceptor that is also partially assimilated as a carbon source [74]. Furthermore, more energy yields are theoretically possible when cells grow via nitrate ammonification than via the reduction of fumarate to succinate (∆*G*^o^’ = 599.6 kJ/mol or 33 kJ/mol, respectively [75]). This ensures greater respiratory gains with the acetate/nitrate pair and faster growth (~2 h generation times compared to >4 h with fumarate) (Fig. 3). Thus, the adaptive specialization of *Geobacterales* for acetate competitively secures an abundant electron donor and carbon source to maximize growth efficiency via DNRA.

The finding that nitrite toxicity is the major rate-limiting step for *T. lovleyi* DNRA is consistent with the more favorable kinetics of nitrate than nitrite reduction in this organism [57] and the preferential diversion of respiratory electrons from nitrite to nitrate reduction reported for other bacteria [63, 64]. We tested acetate and nitrate concentrations covering a wide range of molar acetate/NO_3_^−^ ratios (from 0.3 to 4) (Figs. 4 and 6). DNRA growth was consistently stimulated under conditions that prevented the accumulation of nitrite above toxic (>0.5 mM) concentrations (Fig. 5). Nitrite buildup and cytotoxicity could explain why cells tightly regulated the DNRA respiratory chains, undergoing a reproducible lag phase to acclimate to nitrate even after successive passages of the cells in the same acetate/nitrate medium (Figs. 3 and 4). Acclimation involved the transcriptional upregulation of pathways for the stepwise reduction of nitrate to ammonium via Nap/Nar (reaction 1) and NrfA (reaction 2) proteins (Fig. 3). Increasing nitrate availability (from 5 to 10 mM) further upregulated the respiratory chains did not proportionally increased the enzymatic activities (Fig. 5) to prevent the accumulation of nitrite above toxic levels (Fig. 4). In fact, nitrate reductase activities were repressed at high nitrate concentrations (Fig. 5), which may reflect the feedback inhibition of the enzyme by the nitrite product as the nitrate substrate is rapidly reduced to nitrite. Nitrate concentrations above a certain threshold can also trigger structural changes in the bacterial Nar catalytic subunit that decrease the enzyme’s affinity for the electron acceptor [76]. High nitrate levels can post-transcriptionally downregulate nitrate reductases via small rRNAs as well [77]. Studies with *Citrobacter* and *Enterobacter* spp. isolated from rice paddy soils additionally suggest that nitrate can competitively inhibit the reduction of nitrite to ammonium [78]. Similar mechanisms could have prevented further activation of nitrite reductases for the detoxification of nitrite accumulating in the 10 mM nitrate cultures (Fig. 5). As in other bacteria [63], electrons may have preferentially flown towards the reduction of nitrate, diverting them away from nitrite reductases and exacerbating nitrite toxicity. In support of this, supplying excess electrons via the oxidation of H_2_ stimulated nitrite detoxification and DNRA growth even under conditions of severe acetate limitation [39] (Fig. 6). These results highlight the dual roles that DNRA plays in both nitrate respiration and nitrite detoxification [15, 79–81] and provide a plausible explanation for the prevalence of ammonification over denitrification when nitrate, rather than nitrite, is the dominant electron acceptor for respiration [27]. These findings also underscore the importance of non-carbon electron donors such as H_2_ in supplying electrons for the detoxification of nitrite and modulating DNRA rates.

The widespread presence of nitrite reductases in *Geobacteraceae* (Fig. 2) further highlights key contributions of the group to DNRA via nitrite detoxification. This points at nitrate/nitrite ammonification as an overlooked physiological capability of a group that has been traditionally associated with metal (mainly iron) reduction [37]. Rapid accumulation of Fe(III) (hydr)oxide minerals in DNRA hotspots such as the riverbank of riparian zones [82] and paddy soils impacted by long-term fertilization [83, 84] enriches for *Geobacterales* and promotes the formation of porous iron mineral meshes that concentrate nutrients locally and further stimulate iron reduction [85]. Nitrate readily adsorbs to the iron minerals [86], increasing its local availability for iron reducers. Much of the CO_2_ generated by iron reducers during the oxidation of organic matter in these habitats co-precipitates with Ca^2+^, effectively sequestering the carbon in a stable carbonate mineral sinks [85] and favoring DNRA bacteria with the Ca^2+^-independent pentaheme nitrite reductases of *Geobacterales* [48]. Acetate oxidation with Fe(III) oxides is slightly more favorable than with nitrate (∆G^0^’ = −612 kJ/mol compared to −500 kJ/mol) [87, 88]. However, DNRA has a higher electron-accepting capacity (8 electrons for the nitrate/ammonium pair, compared to 1 electron for Fe(III)/Fe(II)). This favors the use of nitrate over Fe(III) oxides as electron acceptor and the detoxification of nitrite with excess electrons generated during hydrogenotrophic growth (Fig. 6). Abiotic ammonification of nitrite by Fe(II), which is abundantly released during the dissimilatory reduction of Fe(III) (hydr)oxides by *Geobacterales* [89, 90], could further alleviate the physiological constraints imposed by nitrite cytotoxicity. Thus, by intertwining the cycles of C, N and Fe, species such as *T. lovleyi* maximize resource utilization and alleviate growth constraints imposed by nitrite toxicity.

The low metabolic maintenance energy demand of *Geobacterales* species is also of significance to DNRA, because it allows the group to maximize growth yields with otherwise growth-limiting concentrations of both electron donors and acceptors [91]. Microbe-microbe interactions based on nitrite and ammonium cross-feeding could further stimulate DNRA growth with nitrate, and enhance N retention [57]. *T. lovleyi* also acclimated to growth with nitrate by upregulating nitrate sensing networks and genes for flagellar motility and chemotaxis (Table S3). Such regulated response could maximize nitrate foraging and active dispersion from areas with toxic concentrations of nitrite. These and other adaptive responses for DNRA growth revealed in this study provide a plausible explanation for the prevalence of *Geobacteraceae* in DNRA hotspots within riparian corridors [31], where the lateral movement of dissolved nutrients establishes spatial redox gradients that partition DNRA and denitrification [31]. *Geobacteraceae* are also main drivers of DNRA in waterlogged paddy soils [32] and their activities can be stimulated *in situ* via the controlled addition of electron donors or acceptors [92]. This makes the group an attractive target for soil N management via targeted interventions (e.g. control of nitrate inputs [81]) that retain the mobile nitrate species as ammonium and ensure long-term soil nutrition and plant growth. Reports that elevated CO_2_ may favor the uptake of ammonium over nitrate by some commercially important crops such as wheat [93] further emphasize the utility of these microbial strategies for the retention of ammonium species in soils. Self-regulation of the microbial pathways that drive the N cycle and the specialized metabolism of keystone DNRA species affords opportunities to achieve these goals.

## Supplementary Material

Low_nitrate_SI-R3_wraf054

Supplementary_Table_S3_wraf054

Supplementary_Table_S4_wraf054

## Data Availability

The RNA-Seq datasets generated during the current study are available under accession number GSE164776 in the NCBI Gene Expression Omnibus (GEO) repository.
