# Supplementary material for: Adaptive responses of Trichlorobacter lovleyi to nitrite detoxification reveal overlooked contributions of Geobacterales to nitrate ammonification
Source: ISME J. 2025 Mar 18;19(1):wraf054. doi: 10.1093/ismejo/wraf054 (PMC11972089; doi:10.1093/ismejo/wraf054)
Supplement: Low_nitrate_SI-R3_wraf054 [file low_nitrate_si-r3_wraf054.docx]

**Supplemental Material**

**Adaptive responses of *Trichlorobacter lovleyi* to nitrite detoxification reveal overlooked contributions of *Geobacterales* to nitrate ammonification**

Marcela Tabares, Kazem Kashefi, and Gemma Reguera*****

*Department of Microbiology Genetics and Immunology, Michigan State University, East Lansing, Michigan, USA*

*****Corresponding author:

Gemma Reguera

567 Wilson Rd., Rm 6109

Biomedical and Physical Science bld.

Michigan State University

East Lansing, MI 48824

Tel.: (517) 884-5401

e-mail: reguera@msu.edu

**Short title**: Nitrate ammonification in *Geobacterales*

**Keywords:** DNRA, dissimilatory reduction of nitrate to ammonium, global nitrogen conservation, paddy soils, riparian corridors, nitrate reduction, nitrite toxicity

**Supplemental Discussion**

**Transcriptional acclimation of *T. lovleyi* to DNRA**

Genes differentially expressed genes in acetate cultures with 5 mM nitrate over 20 mM fumarate are listed in **Table S3**. A subset of the upregulated genes with predicted roles in nitrate respiration, sensing, foraging and regulation are listed in **Table S4**. Below is an expansive discussion of the respiratory chains upregulated for the reduction of the nitrate substrate and the nitrite intermediate in the DNRA cultures.

1. **Nitrate reduction**

The genome of *T. lovleyi* SZ encodes redundant pathways for the reduction of nitrate to nitrite with (two Nar systems) or without (one Nap system) energy conservation. A model illustrating the predicted organization of these respiratory chains in the cell envelope is shown in **Fig. S1**. All the pathways were upregulated in DNRA cultures together with proteins needed for the secretion of some of the pathway proteins across the inner membrane.

Nap system:

We identified in the genome of *T. lovleyi* SZ a gene cluster with many of the genes typically organized as an operon (*napFDAGHBC*) for the synthesis of the periplasmic Nap nitrate reductase^1^. The cluster encoded all the proteins considered to be essential for the reduction of nitrate to nitrite in the periplasm (**Fig. S1**). The periplasmic fraction of the Nap system is typically isolated as a two-subunit enzyme comprising NapA (a 90 kDa catalytic subunit with a [4Fe4S] cluster as cofactor) and NapB (a 16kDa electron transfer subunit containing two c-type hemes)^2-5^. The operon also includes *napD* (a ‘private chaperone’ involved in maturation of NapA prior to its transfer to the periplasm^6^), and *nap*G and *napH* genes (encoding proteins that interact to form the quinol oxidase complex that channels electrons from both the menaquinol and ubiquinol pool to the NapA protein^7,8^). The *T. lovleyi nap* operon did not encode a canonical NapC, but it included a NapF-like protein (Glov_1060) annotated as a 4Fe-4S ferredoxin iron-sulfur binding domain protein which shared 51% similarity with the *E. coli* NapF. NapC participates in electron transfer to NapAB^9^. In contrast, NapF is essential for assembling the iron-sulfur center of the catalytic subunit of the periplasmic nitrate reductase (NapA)^10^. Thus, NapC and NapF have distinct roles and do not replace each other's functions. This suggests a direct electron transfer between NapG to NapA in *T. lovleyi* (**Fig. S1**).

Nar system:

We identified two clusters with genes needed to express complete Nar systems (Nar-1 and Nar-2) and one additional cluster with only some of the Nar components (Nar-3). The *nar-1* and *nar-2* gene clusters encoded all the proteins needed for the cytoplasmic reduction of nitrate to nitrite. In *E. coli* a four gene operon (narGHJI) encodes the catalytic molybdoprotein (alpha subunit NarG), an iron-sulfur protein (beta subunit NarH), a specific chaperone (delta subunit NarJ), and a diheme cytochrome *b* (gamma-subunit NarI)^11^. The NarGHI complex is anchored to the cytoplasmic side of the inner membrane by NarI to catalyze the reduction of nitrate to nitrite coupled to the generation of a proton motive force (**Fig. S1**). Cytoplasmic NarJ is a chaperone that facilitates interactions between the catalytic molybdoprotein NarG and the molybdenum cofactor biosynthetic proteins (MogA, MoeA, MobA, and MobB)^12^. One of the proteins involved in the biosynthesis of the molybdenum cofactor, MoeA, was also encoded in the *nar-1* cluster (Glov_0210). Also in the *nar-1* cluster was a gene coding for a membrane-bound protein 56% identical and 71% similar to the Nark nitrate/nitrite exchanger of *E. coli* K-12^12^. This transporter takes up nitrate from the periplasm into the cytoplasm for its reduction to nitrite, which the same transporter excretes to the periplasm^13^. We identified downstream of *nark* a gene (Glov_0207) encoding a protein in the Major Facilitator Superfamily (MFS-1), the largest known superfamily of secondary active transporters^14^. This, and the homology between this protein and the nitrate/nitrite transporter NarK of *E. coli* K-12 (25 % identical and 44 % similar), suggests roles as a facilitator of nitrite transport for the Nar system.

The *nar-1* cluster also encoded a NarC *c*-cytochrome (Glov_0202) 30 % similar and 40% identical to the well-characterized NarC protein of *Thermus thermophilus* that is required for the activity of the nitrate reductase complex^11^. This suggests similar roles for NarC in feeding electrons from the menaquinone pool in the inner membrane to the NarGHI complex^15^. The same essential components were conserved in the proteins encoded in the *nar-2* cluster, consistent with redundant functions for the cytoplasmic reduction of nitrate to nitrite and export of the nitrite intermediate to the periplasm (**Fig. S1**).

**2. Nitrite reduction**

*T. lovleyi* SZ contains three nitrite reductase enzymes annotated as NrfA-1 (Glov_1042) , NrfA-2 (Glov_0211) and Nrf-3 (Glov_1061), as well as a membrane-bound NrfH for anchoring pentaheme NrfA proteins to the periplasmic side of the inner membrane (**Fig. S1**). NrfA-1 and NrfH are encoded in an operon conserved among *Geobacterales* and closely related *Desulfuromonadales* (*Desulfuromonas* species) (**Fig. S2**). Both proteins (NrfA-1 and NrfH) have the conserved features of well-studied NrfA and NrfH protein pairs, suggesting functional complementarity as a periplasmic nitrite reductase catalytic subunit that receives electrons from the menaquinol pool via a membrane bound NrfH anchor (**Fig. S1**). NrfA-1 is a pentaheme cytochrome *c* nitrite reductase with four canonical (CXXCH) heme-binding and one non-canonical CXXCK region^16^. The protein crystallizes as a dimer and, like NrfA-2, it contains the divergent motif at the active site of Ca^2+^-independent nitrite reductases^16^. NrfH is a quinol oxidase of the NapC/NirT family with an N-terminal transmembrane domain for membrane anchoring and four canonical (CXXCH) heme-binding sites for electron transfer from the menaquinol pool to NrfA, which completes the reduction of nitrite to ammonium in the periplasm.

NrfA-2 (Glov_0211) was the most highly upregulated *nrfA* gene in DNRA cultures (**Table S4**). It is annotated as a periplasmic ammonia-forming nitrite reductase and shares with NrfA-1 considerable sequence homology (59% identity and 72% similarity) and ancestry (**Fig. 2** in the main article). Like NrfA-1, NrfA-2 is a pentaheme cytochrome *c* nitrite reductase with 4 CXXCH and 1 CXXCK heme-binding motifs. Yet unlike NrfA-1, NrfA-2 is not encoded in an operon with *nrfH* but immediately downstream of the *nar-1* gene cluster (Glov_0202-0210). This genome organization suggests that NrfA-2 could be functionally paired with the Nar system to reduce nitrite to ammonium once it is excreted to the periplasm (**Fig. S1**).

The third NrfA protein of *T. lovleyi* (NrfA-3) is an octaheme cytochrome *c* nitrite reductase (ONR) encoded by a gene immediately downstream (Glov_1061) of the *nap* gene cluster (Glov_1056-1060), suggesting functional pairing for the ammonification of nitrate in the periplasm. NrfA-3 has low sequence homology with NrfA-1 (26% identity and 40% similarity) and NrfA-2 (25% identity and 39% similarity) and forms a separate line of descent (**Fig. 2** in the main article). The heme organization is typical of ONR enzymes, retaining the 4 canonical CXXCH and 1 non-canonical CXXCK heme-binding motifs of pentaheme NrfA proteins but carrying an extended N-terminal region with 3 additional CXXCH motifs. NrfA-3 shares significant sequence homology (42% identity and 58% similarity) with a membrane-bound octaheme nitrite reductase from *Desulfovibrio alkaliphilus*, which catalyzes the reduction of nitrite to ammonium in the periplasm^17^. We identified in NrfA-3 a 25 amino acid long signal peptide for export across the inner membrane but this sequence overlaps with a transmembrane helix domain (amino acids 7 to 26) that could function as membrane anchor (**Fig. S1**). The predicted transmembrane domain (26-amino acid long) is similar in size as the membrane anchor of the *D. alkaliphilus* ONR protein (29 amino acids). Furthermore, as reported for all ONR enzymes, NrfA-3 is more than 530 amino acids long. ONR enzymes have also been reported in sulfur-oxidizing bacteria^18^ and are, together with *nrfAH*, transcriptionally activated and enzymatically active in environments where DNRA was an active process^19^.

**Supplemental Figures**


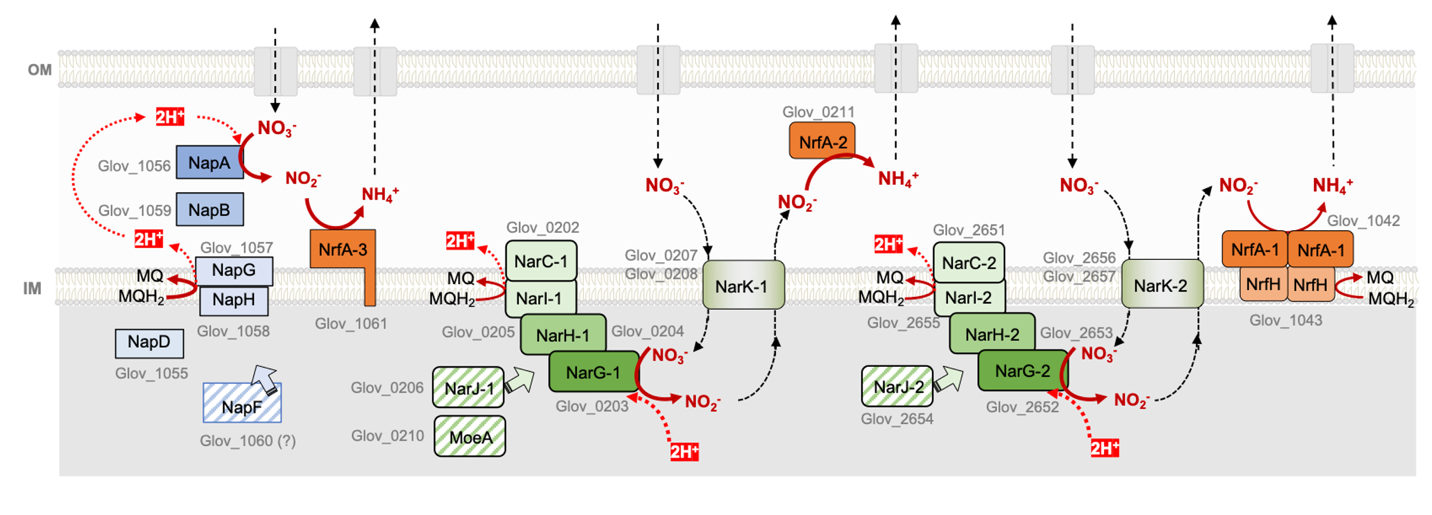


**Figure S1: Reconstruction of the DNRA respiratory chain of *G. lovleyi* SZ.** Proteins in the model were those encoded by genes transcriptionally upregulated in the nitrate over the fumarate cultures and include putative pathways for **1)** the reduction of nitrate (NO_3_^-^) to nitrite (NO_2_^-^) via Nap and Nar nitrate reductase systems (diagonal stripes show proteins predicted to participate in the maturation of the Nap and Nar systems) and **2)** the reduction of the nitrite intermediate to ammonium (NH_4_^+^) via nitrite reductases (NrfA-1 is shown in complex with NrfH; NrfA-2 as a periplasmic enzyme; and NrfA-3 anchored to the periplasmic side of the inner membrane). Reactions generating or consuming protons (H^+^) are shown in red.

**
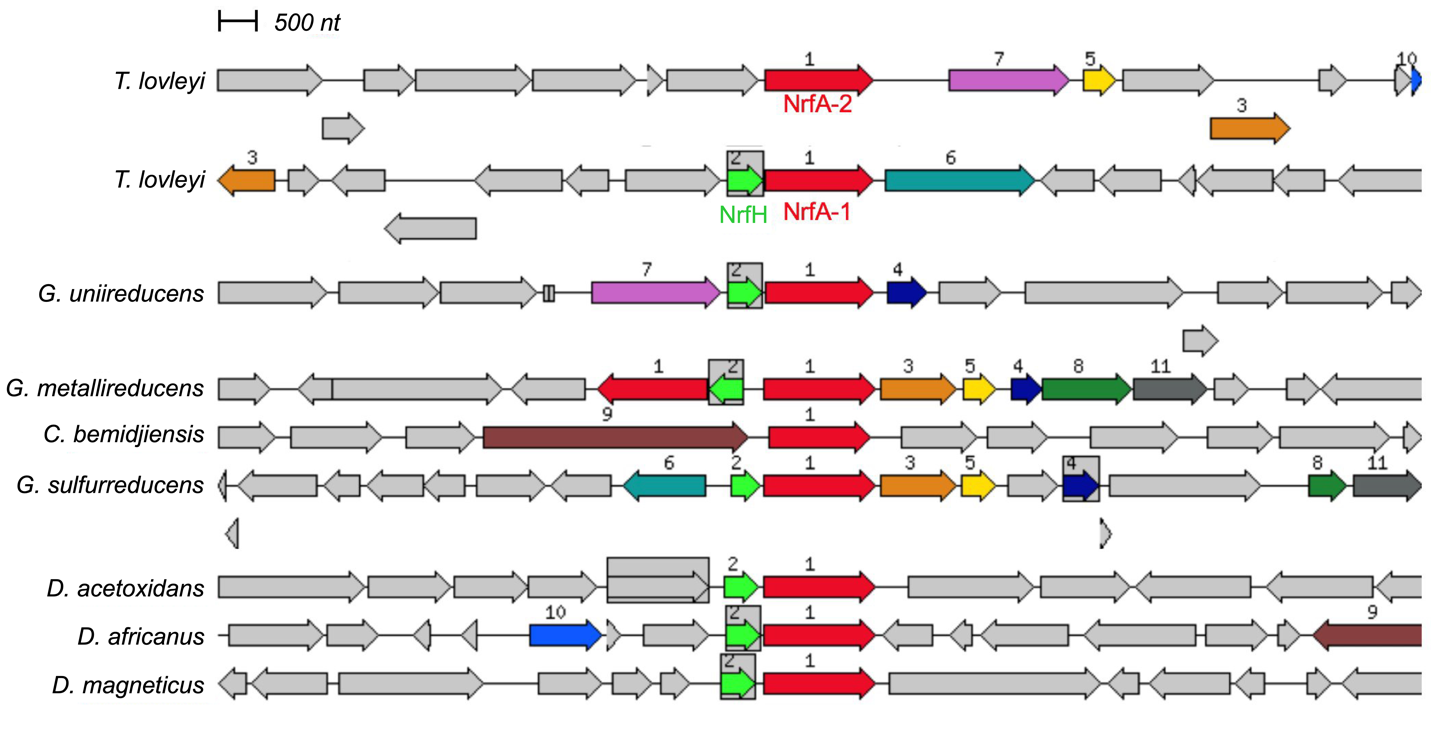
**

**Figure S2: Genomic organization of the *nrfA-1* and *nrfA-2* genes in *T. lovleyi* and homologues in *Geobacterales*.** Comparison of the chromosomal regions around *nrfA-1* and *nrfA-2* genes of *T. lovleyi* and those around the closest *nrfA* homologs in the *Geobacterales* (*Geotalea uraniireducens*, *Geobacter metallireducens, Geobacter sulfurreducens,*  *Citrifermentas bemidjiensis*) and closely related *Desulfuromonadales* (*Desulfuromonas* species). The *nrfA* genes are shown in red and designated with the number 1. The numbers increase based on the frequency of clustering occurrence for genes of the same functional category (2, *nrfH*, in green; 3, hypothetical iron-sulfur cluster binding protein YccM; 4, HPP family protein; 5, PAS/PAC domain protein; 6, methyl-accepting chemotaxis protein; 7, hydroxylamine reductase; 8, molybdopterin-guanine dinucleotide biosynthesis protein MobB; 9, hybrid histidine kinase; 10, ferredoxin 3 fused to uncharacterized domain). Those genes whose relative position is conserved in four or more species (highlighted in gray boxes) are predicted to be functionally coupled. Gene clustering was made using SEED Viewer version 2.0, PubSEED to visualize the organization of the genes surrounding NrfA-1 (Glov_1042) in *T. lovleyi* SZ and its closest homologues in *Geobacterales* and *Desulfuromonadales*.

**
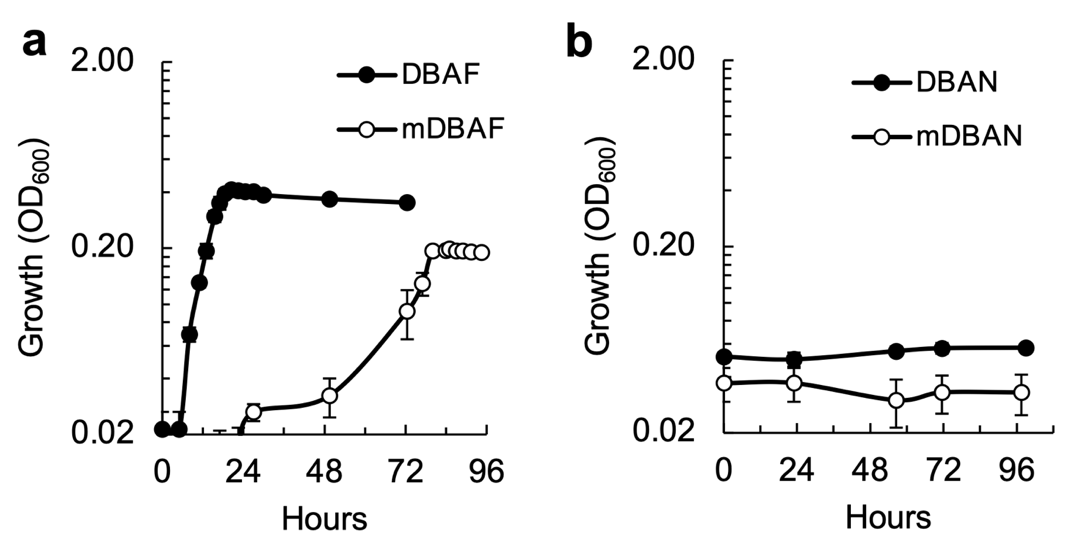
**

**Figure S3: Growth of *T. lovleyi* SZ in modified DB (mDB) medium with acetate and fumarate or nitrate.** Strain SZ was cultured in DB medium and a modified low CaCl_2_ medium (mDB) containing 20 mM acetate as electron donor and 40 mM fumarate (**a**) or 20 mM nitrate (**b**) as electron acceptor. Growth at 30^o^C was monitored as optical density at 600 nm (OD_600_). Cultures with nitrate could not be grown beyond a second transfer (shown).

**Supplemental Tables**

**Table S1. Culture conditions tested for DNRA growth of *G. lovleyi* SZ**^1^

| **Electron donors** | **Electron acceptors** | **CH_3_COO^–^:NO_3_^−^**  (mg per mg N) | **Growth yields** (OD_600_)^2^ |
| --- | --- | --- | --- |
| Acetate (20 mM) | Nitrate (5 mM) | 3.80 | + |
| Acetate (15 mM) | Nitrate (5 mM) | 2.85 | 0.209±0.006 |
| Acetate (10 mM) | Nitrate (5 mM) | 1.90 | 0.223±0.004 |
| Acetate (5 mM) | Nitrate (5 mM) | 0.95 | 0.226±0.026 |
| Acetate (3 mM) | Nitrate (5 mM) | 0.57 | +/– |
| Acetate (3 mM) + H_2_ | Nitrate (5 mM) | 0.57 | + |
| Acetate (1 mM) + H_2_ | Nitrate (5 mM) | 0.19 | – |
| Acetate (3 mM) + formate (10 mM)^3^ | Nitrate (5 mM) | 0.57 | _ |
| Acetate (20 mM)^3^ | Nitrate (5 mM) +Malate (10 mM) | 3.8 | _ |
| Pyruvate (5 mM) | Nitrate (5 mM) | _ | _ |
| Pyruvate (5 mM) + H_2_ | Nitrate (5 mM) | _ | _ |
| Lactate (5 mM) | Nitrate (5 mM) | – | _ |
| Lactate (5 mM) + H_2_ | Nitrate (5 mM) | – | _ |

^1^All cultures were grown in anoxic bicarbonate-based medium (DB) with 2 mM cysteine as reducing agent and variable concentrations of electron donors and/or acceptors. Cultures used for RNA extraction and sequencing are shaded in gray.

^2^Growth yields for cultures after three transfers in the same medium, except for those which could only be passaged once from acetate-fumarate cultures (positive and/or negative symbols indicate growth, partial growth, or no growth).

^3^Supplemented with FeCl_2_ (0.013 mM) as a reducing agent in addition to cysteine (2 mM).

**Table S2. Primers used for RT-qPCR analysis.**

| **Primer name** | **Sequence (5’-3’)** |
| --- | --- |
| *napA*-F | TCAAGAAGGGGGAAGGGGTT |
| *napA*-R | GTGTGCCAATGTTCCAGCAG |
| *narG*-1-F | ACACTTTCCCACACCATTCA |
| *narG*-1-R | CAGCTTCCAATCCTTCACATTTC |
| *narG*-2-F | GGATTACTGCTCTCCCACATAAC |
| *narG*-2-R | AAACCTTCAGTCAGGATGTCAC |
| *nrfA*-1-F | CATCTTCTGGTCGATCTGCTT |
| *nrfA-1*-R | GTACATCCGCTCCGGTTATG |
| *nrfA*-2-F | CAACTCCGCAAACTGCTCAC |
| *nrfA-2*-R | CTTCACGCCTCGGTTGTTTG |
| *nrfA*-3-F | GTGTGGTCAAGCCGATCAATA |
| *nrfA-3*-R | CATCTTGCCCTTGGTGTAGTT |
| *recA*-F | GGCCTGGTTCTCCTACAATAAA |
| *recA*-R | CATCAACCTGGCCTCAATCT |

**Table S3** (provided as an Excel file)

**Differentially expressed genes in nitrate versus fumarate cultures.** Shown are the ID, genome annotation, and Log_2_ FC (fold change) value for each upregulated and downregulated gene in the nitrate over fumarate cultures from **Fig. 3** in the main article.

**Table S4** (provided as an Excel file)

**Upregulated genes with predicted roles in nitrate respiration, sensing, foraging and regulation.**

**Supplemental References**

1 Wang, H., Tseng, C. P. & Gunsalus, R. P. The *nap*F and *nar*G nitrate reductase operons in *Escherichia coli* are differentially expressed in response to submicromolar concentrations of nitrate but not nitrite. *J. Bacteriol.* **181**, 5303-5308 (1999).

2 Berks, B. C., Ferguson, S. J., Moir, J. W. B. & Richardson, D. J. Enzymes and associated electron transport systems that catalyse the respiratory reduction of nitrogen oxides and oxyanions. *Biochim. Biophys. Acta, Bioenerg.* **1232**, 97-173, doi:10.1016/0005-2728(95)00092-5 (1995).

3 Richardson, D. J., Mcewan, A. G., Page, M. D., Jackson, J. B. & Ferguson, S. J. The identification of cytochromesinvolved in the transfer of electrons to the periplasmic NO_3_^-^ reductase of *Rhodobacter capsulatus* and resolution of a solublenitrate reductase-cytochrome-c552 redox complex. *Eur. J. Biochem.* **194**, 263-270, doi:10.1111/j.1432-1033.1990.tb19452.x (1990).

4 Berks, B. C., Richardson, D. J., Reilly, A., Willis, A. C. & Ferguson, S. J. The *napEDABC* gene cluster encoding the periplasmic nitrate reductase system of *Thiosphaera pantotropha.* *Biochem. J.* **309**, 983–9924, doi:10.1042/bj3090983 (1995).

5 Myers, J. M. & Myers, C. R. Role of the tetrahemecytochrome CymA in anaerobic electron transport in cells of *Shewanella putrefaciens* MR-1 with normal levels ofmenaquinone. *J. Bacteriol.* **182**, 67–75, doi:10.1128/JB.182.1.67-75.2000 (2000).

6 Potter, L. C. & Cole, J. A. Essential roles for the products of the *nap*ABCD genes, but not *nap*FGH, inperiplasmic nitrate reduction by *Escherichia coli* K-12. *Biochem. J.* **344**, 69–76, doi:10.1042/0264-6021:3440069 (1999).

7 Brondijk, T. H., Nilavongse, A., Filenko, N., Richardson, D. J. & Cole, J. A. NapGH components of the periplasmic nitrate reductase of *Escherichia coli* K-12: location, topology and physiological roles in quinol oxidation and redox balancing. *Biochem. J.* **379**, 47-55, doi:0.1042/bj20031115. (2004).

8 Brondijk, T. H. C., Fiegen, D., J., R. D. & Cole, J. A. Roles of NapF, NapG and NapH, subunits of the *Escherichia coli* periplasmic nitrate reductase, in ubiquinol oxidation. *Mol. Microbiol.* **44**, 245-255, doi:10.1046/j.1365-2958.2002.02875.x (2002).

9 Simon, J. *,* Gross, R., Einsle, O., Kroneck, P.M.H., Kröger, A., Klimmek, O. A NapC/NirT-type cytochrome *c* (NrfH) is the mediator between the quinone pool and the cytochrome *c* nitrite reductase of *Wolinella succinogenes*. *Mol. Microbiol.* **35**, 686-696, doi:10.1046/j.1365-2958.2000.01742.x (2000).

10 Olmo-Mira, M. F., Gavira, M., Richardson, D. J., Castillo, F., Conrado, M. V., Roldan, M. D. NapF is a cytoplasmic iron-sulfur protein required for Fe-S cluster assembly in the periplasmic nitrate reductase. *J. Biol. Chem.* **279**, 49727-49735, doi:10.1074/jbc.M406502200 (2004).

11 Zafra, O.*,* Ramírez, S., Castán, P., Moreno, R., Cava, F., Vallés, C., *et al.* A cytochrome *c* encoded by the nar operon is required for the synthesis of active respiratory nitrate reductase in *Thermus thermophilus*. *FEBS Lett.* **523**, 99-102, doi:10.1016/S0014-5793(02)02953-8 (2002).

12 Zheng, H., Wisedchaisri, G. & Gonen, T. Crystal structure of a nitrate/nitrite exchanger. *Nature* **497**, 647-651, doi:10.1038/nature12139 (2013).

13 Clegg, S., Yu, F., Griffiths, L. & Cole, J. A. The roles of the polytopic membrane proteins NarK, NarU and NirC in *Escherichia coli* K-12: two nitrate and three nitrite transporters. *Mol. Microbiol.* **44**, 143-155, doi:10.1046/j.1365-2958.2002.02858.x (2002).

14 Drew, D., North, R. A., Nagarathinam, K. & Tanabe, M. Structures and general transport mechanisms by the Major Facilitator Superfamily (MFS). *Chem. Rev.* **121**, 5289-5335, doi:10.1021/acs.chemrev.0c00983 (2021).

15 Moir, J. W. B. in *Reference & Research Book News* Vol. 26 (2011).

16 Campecino, J. *,* Lagishetty, S., Wawrzak, Z., Sosa Alfaro, V., Lehnert, N., Reguera, G.*,et al.* Cytochrome *c* nitrite reductase from the bacterium *Geobacter lovleyi* represents a new NrfA subclass. *J. Biol. Chem.*, doi:10.1074/jbc.RA120.013981 (2020).

17 Thorup, C. *,* Schramm, A., Findlay, A. J., Findlay, A. J., Finster, K. W., Schreiber, L. Disguised as a sulfate reducer: Growth of the Deltaproteobacterium *Desulfurivibrio alkaliphilus* by sulfide oxidation with nitrate. *mBio* **8**, 1-9, doi:10.1128/mbio.00671-17 (2017).

18 Kraft, B., Strous, M. & Tegetmeyer, H. E. Microbial nitrate respiration - Genes, enzymes and environmental distribution. *Biochim. Biophys. Acta* **155**, 104-117, doi:10.1016/j.jbiotec.2010.12.025 (2011).

19 Kraft, B.*,* Tegetmeyer, H.E., Sharma, R., Klotz, M.G., Ferdelman, T. G., Hettich, R. L., *et al.* The environmental controls that govern the end product of bacterial nitrate respiration. *Science* **345**, 676-679, doi:10.1126/science.1254070 (2014).
